# Sociodemographic and Psychosocial Profiles of Multi-Media Use for Risk Communication in the General Population

**DOI:** 10.3390/ijerph191912777

**Published:** 2022-10-06

**Authors:** Samuel Tomczyk, Maxi Rahn, Silke Schmidt

**Affiliations:** Department Health and Prevention, Institute of Psychology, University of Greifswald, 17489 Greifswald, Germany

**Keywords:** crisis communication, disaster management cycle, risk communication, multi-media, public warning

## Abstract

Although disaster research has acknowledged the role of social media in crisis communication, the interplay of new (e.g., mobile apps) and traditional media (e.g., TV, radio) in public warnings has received less attention, particularly from the recipients’ perspective. Therefore, we examined sociodemographic and psychosocial correlates of different types of media use (i.e., traditional, new, mixed) for receiving public warning messages in a population survey (N = 613, 63% female; Mage = 31.56 years). More than two-thirds (68%) reported mixed media use, with 20% relying on new media and 12% on traditional media. Traditional media users were older and reported lower levels of education, while new media users were significantly younger and reported lower trust toward traditional media (i.e., TV). Migrants were more likely to use new but not mixed media. In sum, most participants utilized a mixture of traditional and new media for warning purposes, which has implications for crisis communication. Though, vulnerable populations (e.g., older and less educated participants) mainly rely on traditional media, stressing the need for continued support. Thus, it is paramount to increasingly use mixed methods designs and concurrently examine multiple channels to reflect real-world warning practices and generate ecologically valid results.

## 1. Introduction

Crises such as the ongoing COVID-19 pandemic prove that swift, reliable, and ongoing open and transparent crisis and emergency risk communication (CERC) are paramount to successful disaster management [1,2]. In this context, warning messages are CERC essentials to guide efficient behavioral response and guarantee high compliance in risk governance [3,4]. These warning messages usually comprise information on the source, guidance, time, location, and hazard/consequence to facilitate appropriate action [5,6,7]. To date, many models have been developed to optimize CERC strategies in planning, preparation, and implementation, such as the Protective Action Decision Model (PADM; [8]), the IDEA Model [9], or the communication-human information processing model (C-HIP; [10]). In line with these models, recommendations for successful warning processes consider sources, channels, and contexts, interpersonal and intrapersonal factors. However, the presence and use of multiple concurrent warning channels and their impact on the public have received less attention from scholars, although several studies point to differential media effects.

### 1.1. Use of Multiple Warning Channels for Risk Communication

For instance, newspapers are more credible and more likely to be shared and talked about in crisis communication [11,12], while blogs are more effective in lowering perceived responsibilities than websites and newspapers [13]. The global rise of social media and Internet-based communication as new media has also received attention from the warning community [14]. An increase in social media use during the COVID-19 pandemic, for example, was discussed regarding its positive coping potential (e.g., digitally seeking or expressing social support) and its public health potential (e.g., reaching and informing vast populations) [15,16,17]. Social media and other online media are used to raise public awareness, monitor situational awareness and reactions following crises, and disseminate warning messages.

The scientific literature presents multiple frameworks to integrate such media, for example, social media, into organizational and governmental risk communication (e.g., [18,19]). A recent, comprehensive review of 104 studies [19] identified the core principles of social media use in crisis communication as facilitating dialogue, providing clear, open, and transparent messages from trustworthy sources, developing relationships before and after crisis events (i.e., long-term), and monitoring communication during and after crises to identify needs and resources (e.g., regarding support). However, Eriksson [19] also points out that traditional media (e.g., TV, radio) needs to be prioritized in crisis communication, as it is more accessible and still believed to be more credible and trustworthy [19,20,21]. Therefore, it is paramount to include traditional as well as new media in warning research.

In this respect, the social-mediated crisis communication model [22,23] aims to integrate traditional and new media via social media influencers, followers, and inactives. The authors find that TV is used more often than Facebook to seek information and traditional media is the most continued form of information due to credibility; however, social networks have become more important to monitor progress, access insider information, and check in on family and friends (e.g., [19,20]). In addition, engagement in social media, for instance, posting information or commenting on blogs, is facilitated by newspaper input [24]. This points to the complementarity of media channels. Furthermore, the use of multiple media channels leads to higher exposure and can indicate higher trust [25]. While this research underlines the interdependencies of media channels, it also points to open questions regarding the simultaneous use of multiple media for warning purposes.

### 1.2. Complementarity of Media Channels

To date, mostly (quasi-)experimental studies comparing traditional and new media focused on single channels or media instead of concurrent media use, meaning a comparison of traditional vs. new media use instead of multiuse. However, media richness theory (c. [26]) and experimental evidence comparing different disaster scenarios [24] indicate that using a multitude of channels and modalities (e.g., videos, pictures, charts, texts) leads to a richer experience and increased urgency and compliance in complex and uncertain and unequivocal situations, such as crises or disasters. This is echoed by the current development of more elaborate warnings, including pictures and interactive maps that lead to higher risk perception and preparedness [27,28,29].

The complexity of multi-media use for crisis communication is further illustrated by selective exposure theory (c. [30,31]), which assumes an active audience [32,33] choosing media forms that are most likely to serve the functions that are personally relevant to them. These functions can include informational utility (e.g., political news), social connection (e.g., social networking sites), or affect management (e.g., researching symptoms online to reduce uncertainty and anxiety). Thus, audience predispositions, such as previous positive experiences with specific media forms or the quality of content, shape media choices and can reinforce predispositions [30]. Since its inception, this theory has been expanded into the channel complementarity theory [34,35,36], which also states that channels are not necessarily chosen based on their accessibility but rather their functionality. For example, if traditional media channels (e.g., newspaper, radio) are believed to be the most trustworthy source for financial news, they will be preferred in times of a financial crisis. If social media provides initial information on a terrorist attack, then people will likely seek out social media for more information. In other words, the individual who feels the functional need to consume a specific channel also consumes other channels that perform the same function. Since systematic individual-level differences exist within populations in the consumption of communicative channels, individuals who consume multiple channels that serve a specific functional need are likely to differ systematically from individuals who do not consume the package of communicative channels that do not offer the same specific function. For instance, individuals interested in politics are likely to read political sections of newspapers, follow political news on television, consume political radio, and visit politics-related websites [34]. People who are less interested in politics might not share the same preferences. In the context of risk communication, however, this differentiation of media use has not been explored in as much detail, for example, with regard to audience characteristics. Therefore, we aim to examine sociodemographic and psychosocial correlates of multi-media use, building on channel complementarity theory and previous research on sociodemographic differences between media users.

### 1.3. Sociodemographic Profiles of Multi-Media Use in Risk Communication

While we know that avid users of social media are younger than users of traditional media (e.g., [19,22,23]), previous research also points to other diverse needs in the population, for instance, ethnicity, with Spanish-speaking populations using different media forms than English speaking populations [37]. Multicultural audiences require good planning and multistage addressing and communication styles, such as expressing emotions, understanding a foreign language, and managing channels [38]. Furthermore, persons with high anxiety and hopelessness respond differently to media messages and might also show different patterns of information-seeking behavior, in that anxiety increases information seeking [39], presumably consuming multiple media channels. Therefore, it is important to examine multi-media use for warning purposes in association with user characteristics as well as psychosocial processes to better understand and optimize the warning process in modern times. To add to the literature, this study examines the concurrent use of multiple traditional (TV, radio, newspapers) and new (online news, social media, apps, push notifications) warning media channels and investigates sociodemographic and psychosocial associations with different types of media use (i.e., users of traditional or new media or both).

## 2. Materials and Methods

### 2.1. Sample

Between May and December 2019, German adults (i.e., general population, ages 18 and up) were recruited via flyers and online adverts to participate in a cross-sectional survey on their experiences and expectancies regarding risk and crisis communication (i.a., use of media channels for warning purposes, experiences with warning messages). Flyers were distributed in cafés, shopping centers, and public administration centers to reach a diverse audience. Online adverts were placed on survey platforms for people interested in public opinion surveys and psychological research. The survey was implemented in SoSci Survey. Overall, 621 participants (63% female; mean age = 31.57 (SD = 17.28)) that were at least 18 years of age and fluent in German provided informed consent and then proceeded to complete the questionnaire. Subsequently, they were compensated with 5 Euros or a voucher of the same value. The study procedure was constructed in accordance with the Declaration of Helsinki and was ethically approval by the Ethics Committee of the University Medicine Greifswald (BB 169/18).

### 2.2. Instruments

The survey consisted of several parts focusing on media use in crises, risk perception regarding several hazards, and sociodemographic data. For this study, self-reported currently used media channels to receive warning messages, as well as sociodemographic data, trait anxiety, worry, and trust in media channels, were analyzed.

Participants reported which of the following channels they currently sought out to receive warning messages in times of crisis (1 (yes), 0 (no)): radio, TV, newspaper, online news, social media (e.g., Facebook, Twitter), apps (e.g., specific warning apps), and push notifications (no specific app). In line with previous research, we discerned traditional (TV, radio, newspaper) and new media (online news, social media, apps, push notifications) in our analysis. Participants also stated how much they trusted each of these media channels on a five-point Likert scale from 1 (not at all) to 5 (very).

Sociodemographic data comprised age, sex (1 (male), 2 (female)), level of education (1 (lower secondary, i.e., up to 10 years of secondary education), 2 (higher secondary, i.e., university entry level)), residential area (1 (rural, i.e., up to 5000 inhabitants), 2 (town, i.e., up to 20,000 inhabitants), 3 (small city, i.e., up to 100,000 inhabitants), 4 (city, i.e., more than 100,000 inhabitants)), and country of origin (1 (Germany), 2 (other)).

Anxiety was measured with the trait version of the State Trait Anxiety Inventory [40] by assessing affective states related to trait anxiety in the past two weeks. The scale consists of 20 items rated on a 4-point Likert scale from 1 (almost never) to 4 (almost always) and showed excellent internal consistency (Cronbach’s α = 0.92). Worry was assessed with the ultra-brief version of the Penn State Worry Questionnaire [41], which consists of three items and showed very good internal consistency (Cronbach’s α = 0.86).

### 2.3. Statistical Analysis

Following an inspection of missing data and imputing missing values, we analyzed sociodemographic and psychosocial characteristics of different types of media use via a three-step process. First, we grouped participants into distinct groups (i.e., no media use, traditional media, new media, or both) according to their reported media preferences. Since all but eight participants used at least one of the listed channels, the first group (i.e., no media use) was not included in the analysis, and the participants were excluded due to the very small sample size. Second, we calculated descriptive statistics and comparisons between groups using chi-square tests for categorial variables, ANOVAs with Tukey post hoc tests for continuous variables, and a MANOVA with Tukey post hoc test for trust in warning channels. Third, we calculated multinomial logistic regressions to predict types of media use by sociodemographic data and psychological variables while holding the other variables constant. The residential area was dummy coded for the analysis. We calculated three comparisons (traditional vs. new, traditional vs. mixed, new vs. mixed). For each regression model, Relative Risk Ratios (RRR), including 95% confidence intervals, are reported as effect sizes. Data analysis was performed with Stata 15.1 (StataCorp LLC, College Station, USA). All analyses were based on α = 0.05.

## 3. Results

Overall, eight participants did not report any media used for warning purposes and were excluded from the analysis. Of the remaining 613 participants (63% female; mean age = 31.56 (17.32)), 75 had incomplete data (23 had missing sociodemographic data (between 1 (country of origin) and 17 (residential area)), 52 had missing psychosocial data (between 2 (anxiety) and 26 (trust in push notifications)), albeit with missingness below 5% for each variable. Visual inspection and missing data analysis pointed to missing completely at random; therefore, missing data were imputed via multiple imputations by chained equations [42] using the Stata mi command with 5 imputed data sets and 50 iterations. Categorical data were imputed using ordinal (residential area) and binary logistic regression models (sex, country of origin) and continuous data using linear regression models. All variables were included in the imputation process as described above. Imputation and statistical analyses were performed on pooled data sets (command: mi estimate).

### 3.1. Description of Warning Media Users

According to the analysis, about two-thirds of our sample (n = 414; 67.54%) used a mix of traditional and new media to receive warning messages. Statistical comparisons showed that traditional media users were significantly older, less educated, and lived in smaller residential areas than the other two groups (see Table 1). In addition, users of both traditional and new media lived in more densely populated areas and used online news media most frequently. Trait anxiety and worry did not differ between groups. However, users of new media reported generally lower trust in traditional media than in both other groups, while traditional media users trusted new media significantly less.

### 3.2. Statistical Comparisons of Media Use

The multinomial logistic regression models identified higher age and lower levels of education as consistent predictors of traditional over new media use (see Table 2). Moreover, participants with a migration background were more likely to use new media (RRR = 2.81 [1.11; 7.08]) than traditional media (i.e., TV, radio, and newspaper). Regarding trust, regression models confirmed the descriptive findings by revealing significant associations between trust in online news and apps in users of new and mixed versus traditional media. Neither sex, anxiety, nor worry was significantly associated with any type of media use.

The comparison of new versus mixed media use pointed to additional significant differences regarding age, migration background, residential area, and trust in media channels. Inhabitants of small cities were less likely to use both forms of media compared to rural areas (RRR = 0.47 [0.23; 0.97]). Participants with a migration background were also less likely to combine traditional and new media (RRR = 0.55 [0.33; 0.93]). Higher age (RRR = 1.01 [1.01; 1.03]) and higher trust in TV-based warnings (RRR = 2.24 [1.59; 3.15]) were associated with mixed media use versus new media use.

## 4. Discussion

This study examined sociodemographic and psychosocial associations (i.e., anxiety, worry, media-related trust) of different types of media use for warning purposes in the German population. It extends previous research by explicitly comparing mixed media use (i.e., a combination of traditional and new media for warning purposes) to traditional and new media use. The results show that most participants (about 68% of our sample) use a mixture of traditional (i.e., TV, radio, newspapers) and new media (i.e., online news, apps, social media, and push notifications) to receive public warning messages. However, about 20% rely exclusively on new media, and about 12% rely solely on traditional media.

### 4.1. Profiles of Media Users

Users of new media were significantly younger and less trusting of traditional media, while users of traditional media were older and reported lower educational achievement. Moreover, participants with a migration background (i.e., not born in Germany) were much more likely to use traditional than new media, but they were less likely to use mixed media when compared to new media. Participants who used mixed media were also older than new media users and mostly lived in either rural or metropolitan but not moderately populated areas (i.e., small cities with up to 100,000 inhabitants).

These findings can be connected to the literature in several ways. First, most of our sample reported mixed media use for warning purposes, which is promising for designing warnings: using multiple channels allows for multimodal stimulation, and the implementation of diverse formats (e.g., videos, graphs, texts) can increase urgency, have a higher chance of gathering attention, and consequently, leading to better preparedness [24,25,27,28,29]. A mixture of traditional and new media also guarantees a higher reach of warning messages across the population [19,21], which is particularly important in rural regions and in diverse populations (e.g., with different sociocultural backgrounds). This might explain the significant association between residential areas and multi-media use in our study, with mixed media users being overrepresented in rural regions as opposed to small cities.

Nevertheless, it is important to consider specific preferences of the population when designing warning messages for each medium to successfully connects audience needs, warning message content, and media function to maintain trust and continued communication [30,32,33]. Since higher trust in either traditional or new media was associated with said media use, it can be assumed that positive experiences with each medium build trust and informs future media use (c. [30]). Social media analytics, such as sentiment analysis (e.g., [17]), can also provide additional information about the profiles of social media users and their preferences that can help to tailor warning messages. However, as about a third of our sample reported exclusive use of either traditional or new media, it is important to further explore these groups of media users.

People who preferred traditional media were older and not as well educated as other participants. These observations coincide with the digital divide [43,44,45] that characterizes differences in access and reach (i.e., primary divide) as well as capabilities and habit of use (i.e., secondary divide) of digital technologies and applications. Older people and people with poor education report lower skills and knowledge regarding the use of digital technologies, as well as restricted access to different types of digital technologies, which might also translate into digital warning processes. Hence, our findings indicate a potential digital divide in crisis communication, as well. Participants affected by the digital divide might prefer traditional media such as TV, which is more accessible and less self-directed, thus requiring lower digital skills and literacy. These hypotheses should be considered when developing crisis communication strategies. Since public warnings are meant to galvanize action, the issue of passive media use might be a hindrance. It is necessary to further study aspects of the warning process, for instance, as pointed out in the PADM [8], for different media channels as well as complementary media use to examine these hypotheses. For instance, a preference for more passive media use (e.g., TV) over more active media use (e.g., self-administered warning apps) might be associated with lower attention and arousal and potentially a higher threshold for action.

On the other hand, younger persons reported a strong reliance on new media, with lower levels of trust in traditional media (e.g., TV), which was surprising. While this points toward an active user taking advantage of media and information selection strategies (e.g., [22,32]), it also poses new challenges since social media fosters the spread of incomplete or incorrect information and conspiracy beliefs, also during a crisis and lacks external validation (e.g., due to a lack of trust in traditional media) (e.g., [19,46,47]). Since many public warnings are still broadcast via traditional media (e.g., TV, radio), this finding questions the reach and acceptance of such warnings as well as subsequent compliance in younger generations. The difference in interactivity and customization of new and traditional media also points to challenges when examining new warning channels, such as cell broadcasts. Cell broadcast as a rather novel warning channel (at least in Germany) has the potential to reach a large audience since it is automatically sent to mobile phones without the need to install a warning app. However, without the option of customization, it might also be less appealing to younger users. Therefore, the acceptance and impact of cell broadcasts on different age groups should be examined. In sum, more research is necessary to monitor the development of media use for crisis communication in younger generations and the interplay of media channel use, trust, psychosocial reactions, and warning compliance. Furthermore, migrants relied on new rather than traditional or mixed media, stressing the importance of well-planned and culturally sensitive online communication during crises (e.g., [38]) if this is the main form of media-based communication for this vulnerable group.

Finally, sex, anxiety, and worry were not associated with media use in our analysis. However, it is important that future research investigate samples with elevated levels of anxiety or worry (e.g., persons with a generalized anxiety disorder) to examine the use of warning channels in these vulnerable populations (e.g., [39]). It is also important to investigate differences in the processing of warning messages across different channels when applying mixed media strategies, including different modalities such as acoustic warnings (radio, TV) and text-based warnings (online news, social media), to understand how predispositions such as trust affect warning compliance, particularly in younger generations that seem to distrust traditional media.

### 4.2. Limitations

The study has several limitations that need to be discussed. The study is a cross-sectional analysis of a German population sample. This means that the associations between variables due not imply causality, although they have several implications for warning research and practice. The sample is not representative of the population (e.g., 63% female), which might result in biased estimates, as females often report higher risk perception than males and may be more likely to respond to warning messages (e.g., [8,19]). Moreover, the cross-cultural inquiry is warranted, particularly to examine differences between migrants and persons of different age groups. The study was also limited to pre-selected warning channels and a short, one-item measure of trust in media channels. Thus, it was not possible to assess different facets of trust that have previously been examined in risk research (e.g., [48]), previous experiences with each warning channel, or the use of specific warning channels (e.g., local warning apps). Mixed methods designs could bridge this gap by eliciting user-specific experiences, attitudes, and behaviors and combining them with assessments of standardizes instruments. While social media-focused research has also proposed more elaborate analyses based on machine learning and deep learning to examine user profiles (e.g., [17]), this is not easily applied to traditional and mixed media use, as data on traditional media use is not always available, and a comprehensive assessment of both types of media use is costly. Nevertheless, future research could develop study designs and assessment procedures to facilitate this process and its translation into public warning practice. Finally, while this study presents one possible approach to examining media use, person-centered approaches such as latent class analysis are also promising by identifying group-specific configurations of media use that represent real-world groups of media users instead of a priori defined ones (for examples of applied latent class analysis, see [49,50,51]).

## 5. Conclusions

This study differentiates types of media use for public warning purposes in the general population. While most participants reported mixed media use, considerable subgroups relied exclusively on traditional or new media to receive public warnings. The analysis of traditional, new, and mixed media revealed significant sociodemographic differences between groups that should be considered in crisis communication research and practice. Future studies should investigate group-specific barriers to and facilitators of mixed media use, including structural (e.g., availability and access of different media channels), social (e.g., cultural trust, social norms), and individual (e.g., digital literacy, data security concerns) variables. From a methodological perspective, experimental and field studies using situational and momentary assessments (e.g., ecological momentary assessments) are needed to examine information processing and decision-making processes in relevant scenarios when multiple channels are available and being used (e.g., [52]). This is particularly important for different age groups that differ in their preferred warning channels. In addition, modern technologies such as virtual reality as well as sophisticated online experiments and simulations offer unique opportunities to model and analyze realistic disaster scenarios that allow for observational and interventional research on the use of warning channels and processing of warning messages across diverse groups of the population (e.g., [53,54,55]). Integrative research efforts should also focus on combining sophisticated machine learning and deep learning models of social media use (e.g., [17]) with analyses of traditional media use (e.g., TV, radio) for warning purposes. In sum, while mixed media use for crisis communication seems to become the norm in the general population, it is essential to also acknowledge and monitor different types and trajectories of media use to co-develop adaptive crisis communication strategies and boost public preparedness, particularly regarding vulnerable populations.

## Figures and Tables

**Table 1:** Comparison of mean values and relative frequencies of sociodemographic and psychosocial variables between different groups of media use for warning purposes (i.e., traditional, new, or mixed media) (imputed data set; N = 613).

	Traditional Media (n = 76)	New Media (n = 123)	Traditional + New (n = 414)	Comparisons between Groups ^*^
Sex Female Male	42 (55.26) 34 (44.74)	82 (66.67) 41 (33.33)	265 (64.01) 149 (35.99)	χ^2^ (2) = 2.80, *p* = 0.246
Age	48.39 (26.06)	25.37 (9.01)	30.51 (15.25)	F (2, 580) = 49.09, *p* < 0.001
Level of education Lower secondary Higher secondary	37 (48.68) 39 (51.32)	23 (18.70) 99 (81.30)	80 (19.32) 334 (80.68)	χ^2^ (2) = 32.91, *p* = < 0.001
Residential area Rural Town Small city City	18 (23.68) 18 (23.68) 32 (42.11) 8 (10.53)	15 (12.20) 20 (16.26) 62 (50.41) 26 (21.14)	74 (17.87) 76 (18.36) 156 (37.68) 108 (26.09)	χ^2^ (6) = 15.90, *p* < 0.05
Country of origin Germany Other	62 (81.585) 14 (18.42)	90 (73.17) 33 (26.83)	338 (81.64) 76 (18.36)	χ^2^ (2) = 4.39, *p* = 0.111
Trait anxiety (STAI-T) ^1^	41.08 (10.88)	42.24 (10.43)	40.61 (10.56)	F (2, 580) = 1.08, *p* = 0.340
Worry (PSWQ) ^2^	7.10 (3.28)	7.33 (2.76)	7.21 (2.74)	F (2, 580) = 0.16, *p* = 0.854
Media channels TV Radio Newspaper Online news Social media Apps Push	55 (72.37) 62 (81.58) 32 (42.11) - - - -	- - - 82 (66.67) 76 (61.79) 53 (43.09) 41 (33.33)	306 (73.91) 322 (77.78) 127 (30.68) 331 (79.95) 238 (57.49) 159 (38.41) 98 (23.67)	Two-group comparisons: χ^2^ (1) = 0.08, *p* = 0.779 χ^2^ (1) = 0.55, *p* = 0.459 χ^2^ (1) = 3.83, *p* = 0.050 χ^2^ (1) = 9.42, *p* < 0.01 χ^2^ (1) = 0.72, *p* = 0.395 χ^2^ (1) = 0.87, *p* = 0.351 χ^2^ (1) = 4.61, *p* < 0.05
Trust in media channels ^3^				
TV Radio Newspaper Online news Social media Apps Push	4.16 (1.11) 4.19 (0.95) 3.67 (1.24) 2.94 (1.27) 2.00 (0.99) 2.49 (1.09) 2.65 (1.22)	3.44 (1.13) 3.87 (0.98) 3.34 (1.23) 3.47 (0.90) 2.67 (1.01) 3.32 (1.15) 3.35 (1.26)	4.07 (0.95) 4.12 (0.93) 3.82 (1.01) 3.54 (0.93) 2.50 (0.98) 3.17 (1.11) 3.18 (1.15)	F (2, 610) = 20.32, *p* < 0.001 F (2, 610) = 4.10, *p* < 0.05 F (2, 610) = 9.46, *p* < 0.001 F (2, 610) = 12.48, *p* < 0.001 F (2, 610) = 11.35, *p* < 0.001 F (2, 610) = 14.68, *p* < 0.001 F (2, 610) = 8.67, *p* < 0.001

^*^ significantly different values are underlined; ^1^ STAI-T State Trait Anxiety Inventory—Trait subscale; ^2^ PSWQ Penn State Worry Questionnaire; ^3^ the MANOVA was statistically significant (Roy’s largest root = 0,170; F (7, 605) = 14.73, *p* < 0.001, η_p_^2^ = 0.146).

**Table 2:** Results of the multinomial logistic regression of types of media use (i.e., traditional, new, or mixed media) predicted by sociodemographic (sex, age, education, residential area, country of origin) as well as psychosocial variables (anxiety, worry, trust in media channels) (imputed data set; N = 613).

	New Media vs. Traditional Media *RRR* [95% CI] ^*^	Traditional + New vs. Traditional Media *RRR* [95% CI] ^*^	Traditional + New vs. New Media *RRR* [95% CI] ^*^
Sex (ref. female)	1.46 [0.68; 3.14]	1.16 [0.62; 2.16]	0.79 [0.48; 1.31]
Age	0.94 [0.92; 0.96]	0.96 [0.95; 0.98]	1.01 [1.01; 1.03]
Level of education (ref. lower secondary)	2.59 [1.10; 6.06]	2.43 [1.24; 4.76]	0.94 [0.51; 1.74]
Residential area (ref. rural) Town Small city City	1.04 [0.32; 3.41] 1.55 [0.54; 4.46] 1.39 [0.40; 4.89]	0.81 [0.32; 2.06] 0.73 [0.31; 1.71] 1.24 [0.44; 3.55]	0.77 [0.34; 1.77] 0.47 [0.23; 0.97] 0.89 [0.40; 1.99]
Country of origin (ref. Germany)	2.81 [1.11; 7.08]	1.54 [0.68; 3.49]	0.55 [0.33; 0.93]
Trait anxiety (STAI-T) ^1^	0.99 [0.94; 1.04]	0.98 [0.95; 1.03]	0.99 [0.96; 1.02]
Worry (PSWQ) ^2^	0.99 [0.83; 1.18]	1.05 [0.91; 1.22]	1.07 [0.96; 1.20]
Trust in media channels TV Radio Newspaper Online news Social media Apps Push	0.35 [0.20; 0.61] 1.06 [0.62; 1.81] 0.93 [0.58; 1.49] 2.52 [1.59; 4.00] 1.31 [0.85; 2.02] 1.52 [1.03; 2.23] 1.11 [0.78; 1.58]	0.78 [0.47; 1.27] 0.78 [0.49; 1.26] 1.06 [0.71; 1.58] 2.08 [1.41; 3.06] 1.06 [0.72; 1.54] 1.42 [1.03; 1.95] 0.95 [0.71; 1.28]	2.24 [1.59; 3.15] 0.74 [0.54; 1.01] 1.14 [0.85; 1.53] 0.83 [0.62; 1.11] 0.81 [0.63; 1.04] 0.93 [0.73; 1.19] 0.86 [0.68; 1.08]

* significant values are underlined, CI confidence interval; ^1^ STAI-T State Trait Anxiety Inventory—Trait subscale; ^2^ PSWQ Penn State Worry Questionnaire.

## Data Availability

The data presented in this study are available on request from the corresponding author. The data are not publicly available due to ethical concerns.
